# Supplementary material for: Modeling glioblastoma heterogeneity as a dynamic network of cell states
Source: Mol Syst Biol. 2021 Sep 16;17(9):e10105. doi: 10.15252/msb.202010105 (PMC8444284; doi:10.15252/msb.202010105)
Supplement: Supplementary file 5 — Source Data for Figure 3 [file MSB-17-e10105-s001.zip › Figure3A_sourcedata/GSEA_3065/hallmarks_state2.GseaPreranked.1623416269049/HALLMARK_IL2_STAT5_SIGNALING.html]

Details for gene set HALLMARK\_IL2\_STAT5\_SIGNALING[GSEA]

|  || Dataset | state2 |
| Phenotype | NoPhenotypeAvailable |
| Upregulated in class | na\_neg |
| GeneSet | HALLMARK\_IL2\_STAT5\_SIGNALING |
| Enrichment Score (ES) | -0.46814764 |
| Normalized Enrichment Score (NES) | -1.8154813 |
| Nominal p-value | 0.0 |
| FDR q-value | 0.0018281038 |
| FWER p-Value | 0.01 |
Table: GSEA Results Summary

  

Fig 1: Enrichment plot: HALLMARK\_IL2\_STAT5\_SIGNALING      
 Profile of the Running ES Score & Positions of GeneSet Members on the Rank Ordered List

  

| PROBE | GENE SYMBOL | GENE\_TITLE | RANK IN GENE LIST | RANK METRIC SCORE | RUNNING ES | CORE ENRICHMENT || 1 | CDC6 |  |  | 106 | 0.372 | 0.0282 | No |
| 2 | CCND2 |  |  | 227 | 0.263 | 0.0433 | No |
| 3 | ITGAE |  |  | 292 | 0.236 | 0.0616 | No |
| 4 | GPX4 |  |  | 425 | 0.194 | 0.0680 | No |
| 5 | GSTO1 |  |  | 884 | 0.130 | 0.0328 | No |
| 6 | CCNE1 |  |  | 888 | 0.129 | 0.0462 | No |
| 7 | AHCY |  |  | 959 | 0.123 | 0.0518 | No |
| 8 | UMPS |  |  | 1145 | 0.109 | 0.0435 | No |
| 9 | CCND3 |  |  | 1164 | 0.107 | 0.0529 | No |
| 10 | ETV4 |  |  | 1840 | 0.071 | -0.0119 | No |
| 11 | PLSCR1 |  |  | 1853 | 0.070 | -0.0057 | No |
| 12 | NCS1 |  |  | 1870 | 0.070 | 0.0000 | No |
| 13 | ODC1 |  |  | 1913 | 0.068 | 0.0027 | No |
| 14 | DCPS |  |  | 1953 | 0.066 | 0.0056 | No |
| 15 | CASP3 |  |  | 2028 | 0.064 | 0.0045 | No |
| 16 | UCK2 |  |  | 2073 | 0.062 | 0.0063 | No |
| 17 | CA2 |  |  | 2111 | 0.061 | 0.0088 | No |
| 18 | BATF3 |  |  | 2348 | 0.052 | -0.0109 | No |
| 19 | ST3GAL4 |  |  | 2391 | 0.051 | -0.0100 | No |
| 20 | BCL2 |  |  | 2499 | 0.048 | -0.0164 | No |
| 21 | ARL4A |  |  | 2510 | 0.047 | -0.0124 | No |
| 22 | PHTF2 |  |  | 2622 | 0.044 | -0.0196 | No |
| 23 | SPRY4 |  |  | 2656 | 0.043 | -0.0186 | No |
| 24 | GLIPR2 |  |  | 2669 | 0.043 | -0.0154 | No |
| 25 | RHOB |  |  | 2690 | 0.042 | -0.0130 | No |
| 26 | RNH1 |  |  | 2962 | 0.035 | -0.0384 | No |
| 27 | HIPK2 |  |  | 3029 | 0.033 | -0.0419 | No |
| 28 | DENND5A |  |  | 3135 | 0.031 | -0.0499 | No |
| 29 | CDC42SE2 |  |  | 3168 | 0.030 | -0.0501 | No |
| 30 | CAPG |  |  | 3281 | 0.028 | -0.0591 | No |
| 31 | MYC |  |  | 3842 | 0.018 | -0.1171 | No |
| 32 | PUS1 |  |  | 4015 | 0.015 | -0.1340 | No |
| 33 | RRAGD |  |  | 4059 | 0.015 | -0.1370 | No |
| 34 | NFIL3 |  |  | 4213 | 0.012 | -0.1521 | No |
| 35 | MAPKAPK2 |  |  | 4390 | 0.009 | -0.1700 | No |
| 36 | SNX9 |  |  | 4485 | 0.008 | -0.1793 | No |
| 37 | EEF1AKMT1 |  |  | 4574 | 0.006 | -0.1881 | No |
| 38 | SPRED2 |  |  | 4630 | 0.005 | -0.1934 | No |
| 39 | PDCD2L |  |  | 4666 | 0.005 | -0.1967 | No |
| 40 | PLIN2 |  |  | 4837 | 0.002 | -0.2147 | No |
| 41 | SLC39A8 |  |  | 4873 | 0.001 | -0.2183 | No |
| 42 | SWAP70 |  |  | 5075 | -0.001 | -0.2397 | No |
| 43 | PTRH2 |  |  | 5184 | -0.003 | -0.2509 | No |
| 44 | IKZF4 |  |  | 5389 | -0.007 | -0.2721 | No |
| 45 | MYO1C |  |  | 5468 | -0.008 | -0.2796 | No |
| 46 | MAP6 |  |  | 5527 | -0.009 | -0.2849 | No |
| 47 | SLC1A5 |  |  | 5584 | -0.010 | -0.2898 | No |
| 48 | RABGAP1L |  |  | 5747 | -0.012 | -0.3059 | No |
| 49 | FAH |  |  | 5837 | -0.014 | -0.3139 | No |
| 50 | HOPX |  |  | 6156 | -0.019 | -0.3460 | No |
| 51 | LCLAT1 |  |  | 6182 | -0.020 | -0.3466 | No |
| 52 | CYFIP1 |  |  | 6368 | -0.023 | -0.3639 | No |
| 53 | SERPINB6 |  |  | 6465 | -0.025 | -0.3716 | No |
| 54 | FURIN |  |  | 6544 | -0.026 | -0.3772 | No |
| 55 | NCOA3 |  |  | 6632 | -0.028 | -0.3835 | No |
| 56 | POU2F1 |  |  | 6633 | -0.028 | -0.3806 | No |
| 57 | COCH |  |  | 6724 | -0.030 | -0.3870 | No |
| 58 | HUWE1 |  |  | 6860 | -0.033 | -0.3980 | No |
| 59 | PHLDA1 |  |  | 6867 | -0.033 | -0.3951 | No |
| 60 | PRAF2 |  |  | 6920 | -0.035 | -0.3970 | No |
| 61 | XBP1 |  |  | 6931 | -0.035 | -0.3943 | No |
| 62 | TIAM1 |  |  | 6948 | -0.035 | -0.3923 | No |
| 63 | KLF6 |  |  | 7010 | -0.037 | -0.3949 | No |
| 64 | BCL2L1 |  |  | 7048 | -0.038 | -0.3949 | No |
| 65 | PTCH1 |  |  | 7258 | -0.044 | -0.4126 | No |
| 66 | TWSG1 |  |  | 7335 | -0.045 | -0.4159 | No |
| 67 | ENPP1 |  |  | 7412 | -0.048 | -0.4190 | No |
| 68 | RGS16 |  |  | 7474 | -0.050 | -0.4202 | No |
| 69 | CDKN1C |  |  | 7524 | -0.051 | -0.4200 | No |
| 70 | FAM126B |  |  | 7533 | -0.051 | -0.4154 | No |
| 71 | NDRG1 |  |  | 7538 | -0.051 | -0.4104 | No |
| 72 | GABARAPL1 |  |  | 7630 | -0.054 | -0.4144 | No |
| 73 | SYNGR2 |  |  | 7633 | -0.054 | -0.4088 | No |
| 74 | IFNGR1 |  |  | 7945 | -0.065 | -0.4353 | No |
| 75 | SNX14 |  |  | 8001 | -0.067 | -0.4340 | No |
| 76 | ANXA4 |  |  | 8188 | -0.077 | -0.4458 | No |
| 77 | P4HA1 |  |  | 8398 | -0.089 | -0.4587 | Yes |
| 78 | SLC2A3 |  |  | 8419 | -0.090 | -0.4512 | Yes |
| 79 | CKAP4 |  |  | 8425 | -0.091 | -0.4421 | Yes |
| 80 | SOCS2 |  |  | 8482 | -0.094 | -0.4381 | Yes |
| 81 | AHR |  |  | 8513 | -0.097 | -0.4310 | Yes |
| 82 | CSF1 |  |  | 8560 | -0.101 | -0.4252 | Yes |
| 83 | ADAM19 |  |  | 8683 | -0.110 | -0.4266 | Yes |
| 84 | IFITM3 |  |  | 8720 | -0.113 | -0.4184 | Yes |
| 85 | BMPR2 |  |  | 8774 | -0.119 | -0.4114 | Yes |
| 86 | BHLHE40 |  |  | 8804 | -0.123 | -0.4015 | Yes |
| 87 | ITGA6 |  |  | 8820 | -0.125 | -0.3897 | Yes |
| 88 | PLPP1 |  |  | 8821 | -0.125 | -0.3764 | Yes |
| 89 | APLP1 |  |  | 8911 | -0.137 | -0.3713 | Yes |
| 90 | PRNP |  |  | 9068 | -0.161 | -0.3709 | Yes |
| 91 | IGF2R |  |  | 9107 | -0.167 | -0.3572 | Yes |
| 92 | ALCAM |  |  | 9132 | -0.173 | -0.3414 | Yes |
| 93 | IGF1R |  |  | 9152 | -0.178 | -0.3245 | Yes |
| 94 | EMP1 |  |  | 9156 | -0.179 | -0.3058 | Yes |
| 95 | WLS |  |  | 9168 | -0.181 | -0.2877 | Yes |
| 96 | AHNAK |  |  | 9183 | -0.185 | -0.2695 | Yes |
| 97 | SYT11 |  |  | 9197 | -0.189 | -0.2508 | Yes |
| 98 | NT5E |  |  | 9208 | -0.194 | -0.2312 | Yes |
| 99 | PLEC |  |  | 9220 | -0.200 | -0.2111 | Yes |
| 100 | TNFRSF21 |  |  | 9223 | -0.202 | -0.1898 | Yes |
| 101 | LRIG1 |  |  | 9229 | -0.204 | -0.1686 | Yes |
| 102 | ITGAV |  |  | 9346 | -0.269 | -0.1525 | Yes |
| 103 | CD81 |  |  | 9356 | -0.281 | -0.1235 | Yes |
| 104 | CD44 |  |  | 9407 | -0.397 | -0.0867 | Yes |
| 105 | COL6A1 |  |  | 9410 | -0.410 | -0.0433 | Yes |
| 106 | SPP1 |  |  | 9415 | -0.436 | 0.0027 | Yes |
Table: GSEA details [plain text format]

  

Fig 2: HALLMARK\_IL2\_STAT5\_SIGNALING: Random ES distribution      
 Gene set null distribution of ES for **HALLMARK\_IL2\_STAT5\_SIGNALING**
